# Dredging in the Spratly Islands: Gaining Land but Losing Reefs

**DOI:** 10.1371/journal.pbio.1002422

**Published:** 2016-03-31

**Authors:** Camilo Mora, Iain R. Caldwell, Charles Birkeland, John W. McManus

**Affiliations:** 1 Department of Geography, University of Hawaii at Manoa, Honolulu, Hawaii, United States of America; 2 Hawaii Institute of Marine Biology, University of Hawaii at Manoa, Honolulu, Hawaii, United States of America; 3 Department of Biology, University of Hawaii at Manoa, Honolulu, Hawaii, United States of America; 4 Department of Marine Biology and Ecology, Rosenstiel School, University of Miami, Miami, Florida, United States of America

## Abstract

Coral reefs on remote islands and atolls are less exposed to direct human stressors but are becoming increasingly vulnerable because of their development for geopolitical and military purposes. Here we document dredging and filling activities by countries in the South China Sea, where building new islands and channels on atolls is leading to considerable losses of, and perhaps irreversible damages to, unique coral reef ecosystems. Preventing similar damage across other reefs in the region necessitates the urgent development of cooperative management of disputed territories in the South China Sea. We suggest using the Antarctic Treaty as a positive precedent for such international cooperation.

## Abstract 2

The degradation of unique natural assets in disputed territories calls for radical multinational approaches to conservation.

Coral reefs constitute one of the most diverse, socioeconomically important, and threatened ecosystems in the world [1–3]. Coral reefs harbor thousands of species [4] and provide food and livelihoods for millions of people while safeguarding coastal populations from extreme weather disturbances [2,3]. Unfortunately, the world’s coral reefs are rapidly degrading [1–3], with ~19% of the total coral reef area effectively lost [3] and 60% to 75% under direct human pressures [3,5,6]. Climate change aside, this decline has been attributed to threats emerging from widespread human expansion in coastal areas, which has facilitated exploitation of local resources, assisted colonization by invasive species, and led to the loss and degradation of habitats directly and indirectly through fishing and runoff from agriculture and sewage systems [1–3,5–7]. In efforts to protect the world’s coral reefs, remote islands and atolls are often seen as reefs of “hope,” as their isolation and uninhabitability provide de facto protection against direct human stressors, and may help impacted reefs through replenishment [5,6]. Such isolated reefs may, however, still be vulnerable because of their geopolitical and military importance (e.g., allowing expansion of exclusive economic zones and providing strategic bases for military operations). Here we document patterns of reclamation (here defined as creating new land by filling submerged areas) of atolls in the South China Sea, which have resulted in considerable loss of coral reefs. We show that conditions are ripe for reclamation of more atolls, highlighting the need for international cooperation in the protection of these atolls before more unique and ecologically important biological assets are damaged, potentially irreversibly so.

Studies of past reclamations and reef dredging activities have shown that these operations are highly deleterious to coral reefs [8,9]. First, reef dredging affects large parts of the surrounding reef, not just the dredged areas themselves. For example, 440 ha of reef was completely destroyed by dredging on Johnston Island (United States) in the 1960s, but over 2,800 ha of nearby reefs were also affected [10]. Similarly, at Hay Point (Australia) in 2006 there was a loss of coral cover up to 6 km away from dredging operations [11]. Second, recovery from the direct and indirect effects of dredging is slow at best and nonexistent at worst. In 1939, 29% of the reefs in Kaneohe Bay (United States) were removed by dredging, and none of the patch reefs that were dredged had completely recovered 30 years later [12]. In Castle Harbour (Bermuda), reclamation to build an airfield in the early 1940s led to limited coral recolonization and large quantities of resuspended sediments even 32 years after reclamation [13]; several fish species are claimed extinct as a result of this dredging [14,15]. Such examples and others led Hatcher et al. [8] to conclude that dredging and land clearing, as well as the associated sedimentation, are possibly the most permanent of anthropogenic impacts on coral reefs.

The impacts of dredging for the Spratly Islands are of particular concern because the geographical position of these atolls favors connectivity via stepping stones for reefs over the region [16–19] and because their high biodiversity works as insurance for many species. In an extensive review of the sparse and limited data available for the region, Hughes et al. [20] showed that reefs on offshore atolls in the South China Sea were overall in better condition than near-shore reefs. For instance, by 2004 they reported average coral covers of 64% for the Spratly Islands and 68% for the Paracel Islands. By comparison, coral reefs across the Indo-Pacific region in 2004 had average coral covers below 25% [21]. Reefs on isolated atolls can still be prone to extensive bleaching and mortality due to global climate change [22] and, in the particular case of atolls in the South China Sea, the use of explosives and cyanine [20]. However, the potential for recovery of isolated reefs to such stressors is remarkable. Hughes et al. [20] documented, for instance, how coral cover in several offshore reefs in the region declined from above 80% in the early 1990s to below 6% by 1998 to 2001 (due to a mixture of El Niño and damaging fishing methods that make use of cyanine and explosives) but then recovered to 30% on most reefs and up to 78% in some reefs by 2004–2008. Another important attribute of atolls in the South China Sea is the great diversity of species. Over 6,500 marine species are recorded for these atolls [23], including some 571 reef coral species [24] (more than half of the world’s known species of reef-building corals). The relatively better health and high diversity of coral reefs in atolls over the South China Sea highlights the uniqueness of such reefs and the important roles they may play for reefs throughout the entire region. Furthermore, these atolls are safe harbor for some of the last viable populations of highly threatened species (e.g., Bumphead Parrotfish [*Bolbometopon muricatum*] and several species of sawfishes [*Pristis*, *Anoxypristis*]), highlighting how dredging in the South China Sea may threaten not only species with extinction but also the commitment by countries in the region to biodiversity conservation goals such as the Convention of Biological Diversity Aichi Targets and the United Nations Sustainable Development Goals.

Recently available remote sensing data (i.e., Landsat 8 Operational Land Imager and Thermal Infrared Sensors Terrain Corrected images) allow quantification of the sharp contrast between the gain of land and the loss of coral reefs resulting from reclamation in the Spratly Islands (Fig 1). For seven atolls recently reclaimed by China in the Spratly Islands (names provided in Fig 1D, Table 1), we extracted one cloud-free image for each 60-day period from February 2014 to May 2015. In these images, only land above sea level is visible in the short-wave infrared band (i.e., Landsat band 6), while land above sea level and natural reef areas (e.g., coral reefs and submerged natural sand bars) are both visible in the red optical band (i.e., Landsat band 4). By subtracting the size of visible areas in Landsat band 6 from the size of visible areas in Landsat band 4, we were able to quantify the total size of natural reef areas (see S1 Data for details); the area of reclamation is the size of visible areas in Landsat band 6, as prior to reclamation most of the atolls were submerged, with the exception of small areas occupied by a handful of buildings on piers (note that the amount of land area was near zero at the start of the reclamation; Fig 1C, S1 Data). The seven reclaimed atolls have effectively lost ~11.6 km^2^ (26.9%) of their reef area for a gain of ~10.7 km^2^ of land (i.e., >75 times increase in land area) from February 2014 to May 2015 (Fig 1C). The area of land gained was smaller than the area of reef lost because reefs were lost not only through land reclamation but also through the deepening of reef lagoons to allow boat access (Fig 1B). Similar quantification of reclamation by other countries in the South China Sea (Table 1) was not possible with available Landsat 8 images because reclamation in many of these atolls has occurred prior to the launching of the Landsat 8 satellite in 2013 and because historically there was land above sea level, which precludes differentiating reclaimed land from natural land.

**Fig 1:**
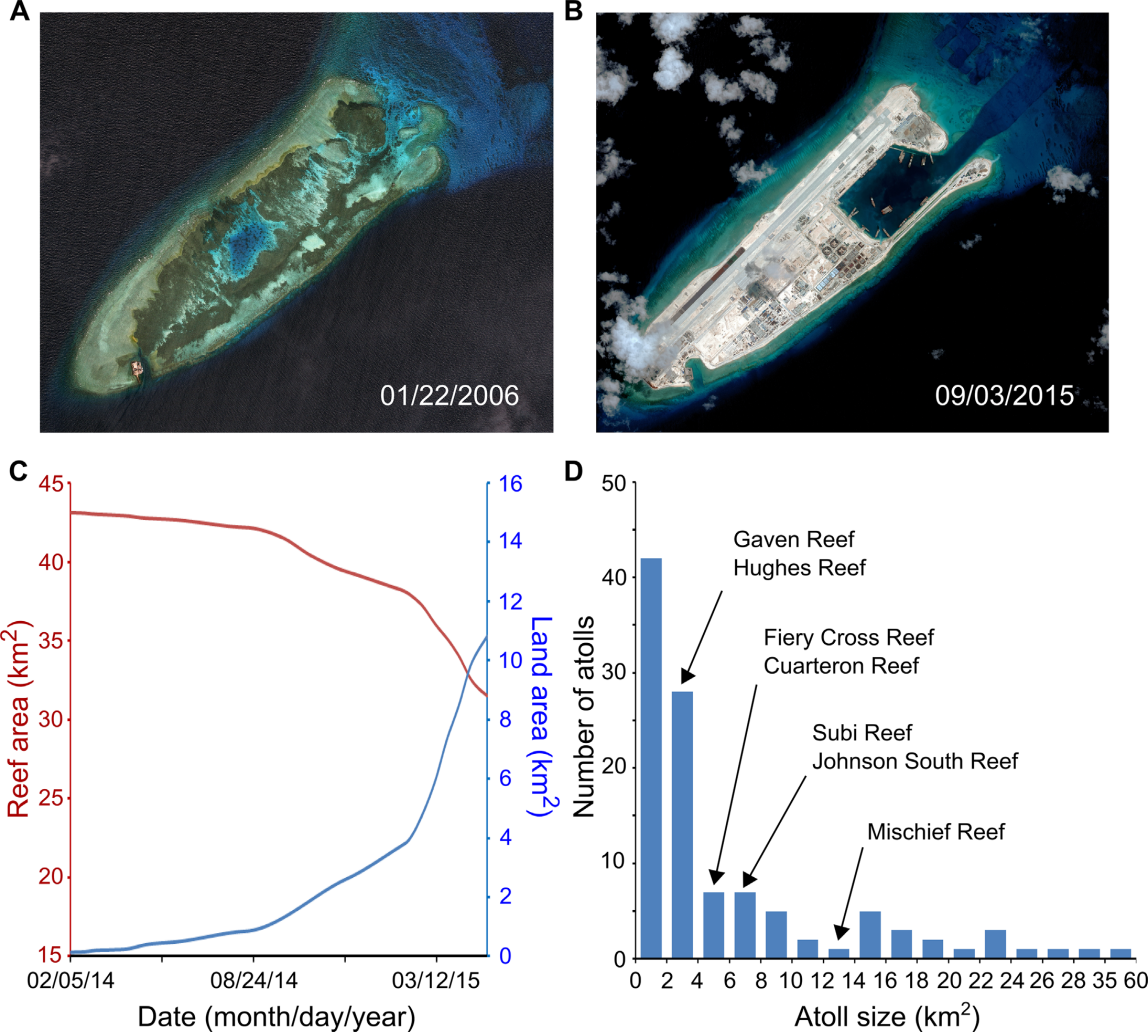
Reclamation leads to gains of land in return for losses of coral reefs: A case example of China’s recent reclamation in the Spratly Islands. For display purposes, we show two images of Fiery Cross Reef before (A) and after (B) land reclamation (images courtesy of the Asia Maritime Transparency Initiative from the Center for Strategic and International Studies and Digital Globe). The cumulative reclamation in the seven atolls has resulted in considerable increases in land (blue line, C) but reductions in coral reef area (red line, C). Changes in land and reefs, over time, for the individual atolls are shown in S2 Data. The Spratly Islands, South China Sea, are rich in atolls with similar sizes and characteristics to those already reclaimed (D, China’s seven recently reclaimed atolls are highlighted with arrows in their respective size categories). Data for plots C–D are provided in S2–S4 Data. Quantifying similar trends for the reclamation of other atolls by other countries was not possible with available Landsat 8 images because reclamation in many of these atolls had occurred prior to the launching of the Landsat 8 satellite in 2013 and because historically there was land above sea level, which precludes differentiating reclaimed land from natural land.

**Table 1:** List of reclaimed atolls in the Spratly Islands and the Paracel Islands. Several countries are responsible for the land fillings but are not named to avoid implying ownership.

SPRATLY ISLANDS	Latitude	Longitude
Cuarteron Reef	8°51ʹ39.04ʺN	112°50ʹ20.52ʺE
Fiery Cross Reef	9°32ʹ53.33ʺN	112°53ʹ18.59ʺE
Gaven Reef	10°12ʹ29.25ʺN	114°13ʹ22.52ʺE
Hughes Reef	9°54ʹ51.29ʺN	114°29ʹ51.57ʺE
Johnson South Reef	9°43ʹ11.81ʺN	114°16ʹ56.30ʺE
Mischief Reef	9°54ʹ8.19ʺN	115°32ʹ14.22ʺE
Subi Reef	10°55ʹ31.53ʺN	114°5ʹ6.03ʺE
Erica Reef	8°6ʹ27.29ʺN	114°8ʹ1.88ʺE
Mariveles Reef	7°58ʹ3.09ʺN	113°55ʹ13.54ʺE
Swallow Reef	7°22ʹ28.80ʺN	113°49ʹ43.79ʺE
Thitu Island	11°3ʹ13.87ʺN	114°17ʹ5.89ʺE
Itu Aba Island	10°22ʹ37.36ʺN	114°21ʹ56.44ʺE
Central Reef	8°55ʹ51.13ʺN	112°21ʹ0.47ʺE
Namyit Island	10°10ʹ46.13ʺN	114°21ʹ57.63ʺE
Pearson Reefs	8°57ʹ28.47ʺN	113°40ʹ38.21ʺE
Sand Cay	10°22ʹ28.72ʺN	114°28ʹ48.63ʺE
Sin Cowe Island	9°53ʹ7.52ʺN	114°19ʹ47.29ʺE
Southwest Cay	11°25ʹ45.36ʺN	114°19ʹ54.05ʺE
Spratly Island	8°38ʹ42.03ʺN	111°55ʹ13.15ʺE
West Reef	8°51ʹ45.58ʺN	112°13ʹ29.83ʺE
**PARACEL ISLANDS**		
Duncan Island	16°27ʹ6.41ʺN	111°42ʹ37.06ʺE
Lincoln Island	16°39ʹ59.93ʺN	112°43ʹ49.44ʺE
Money Island	16°26ʹ51.70ʺN	111°30ʹ25.13ʺE
Palm Island	16°27ʹ8.01ʺN	111°42ʹ2.62ʺE
Pattle Island	16°32ʹ2.76ʺN	111°36ʹ25.93ʺE
Rocky Island	16°50ʹ39.71ʺN	112°20ʹ50.41ʺE
Triton Island	15°47ʹ6.02ʺN	111°12ʹ15.13ʺE
Woody Island	16°50ʹ4.82ʺN	112°20ʹ15.70ʺE

The impacts of reclamation on coral reefs are likely more severe than simple changes in area, as reclamation is being achieved by means of suction dredging (i.e., cutting and sucking materials from the seafloor and pumping them over land). With this method, reefs are ecologically degraded and denuded of their structural complexity. Dredging and pumping also disturbs the seafloor and can cause runoff from reclaimed land, which generates large clouds of suspended sediment [11] that can lead to coral mortality by overwhelming the corals’ capacity to remove sediments and leave corals susceptible to lesions and diseases [7,9,25]. The highly abrasive coralline sands in flowing water can scour away living tissue on a myriad of species and bury many organisms beyond their recovery limits [26]. Such sedimentation also prevents new coral larvae from settling in and around the dredged areas, which is one of the main reasons why dredged areas show no signs of recovery even decades after the initial dredging operations [9,12,13]. Furthermore, degradation of wave-breaking reef crests, which make reclamation in these areas feasible, will result in a further reduction of coral reefs’ ability to (1) self-repair and protect against wave abrasion [27,28] (especially in a region characterized by typhoons) and (2) keep up with rising sea levels over the next several decades [29]. This suggests that the new islands would require periodic dredging and filling, that these reefs may face chronic distress and long-term ecological damage, and that reclamation may prove economically expensive and impractical.

The potential for land reclamation on other atolls in the Spratly Islands is high, which necessitates the urgent development of cooperative management of disputed territories in the South China Sea. First, the Spratly Islands are rich in atolls with similar characteristics to those already reclaimed (Fig 1D); second, there are calls for rapid development of disputed territories to gain access to resources and increase sovereignty and military strength [30]; and third, all countries with claims in the Spratly Islands have performed reclamation in this archipelago (Table 1; at least 20 atolls have been reclaimed in the Spratly Islands, and this does not include reclamation activities in the Paracel Islands). In the Spratly Islands, where no country can gain full access to resources without generating international conflict and where the race for development could cause irreversible damage to unique natural assets, novel multinational approaches to conservation are urgently needed [20]. One such possibility is the generation of a multinational marine protected area [16,17]. Such a marine protected area could safeguard an area of high biodiversity and importance to genetic connectivity in the Pacific, in addition to promoting peace in the region (extended justification provided by McManus [16,17]). A positive precedent for the creation of this protected area is that of Antarctica, which was also subject to numerous overlapping claims and where a recently renewed treaty froze national claims, preventing large-scale ecological damage while providing environmental protection and areas for scientific study. Development of such a legal framework for the management of the Spratly Islands could prevent conflict, promote functional ecosystems, and potentially result in larger gains (through spillover, e.g. [31]) for all countries involved.

## Supporting Information

S1 Data Methods used to quantify the area of reefs dredged and filled in the Spratly Islands using Landsat 8 imagery. (PDF)

S2 Data Raw and interpolated data from Landsat 8 imagery (as shown in Fig 1C). (XLSX)

S3 Data Sizes of atolls in the Spratly Islands (data shown in Fig 1D). (XLSX)

S4 Data Compressed folder containing the shapefiles created from Landsat 8 imagery to calculate changes in land and reef areas over time for seven recently reclaimed atolls in the Spratly Islands. (ZIP)

## References

[1] Bellwood DR, Hughes TP, Folke C, Nystrom M. Confronting the coral reef crisis. Nature 2004; 429: 827–833. 15215854 10.1038/nature02691

[2] Burke L, Reytar K, Spalding M, Perry A. Reefs at risk revisited World Resources Institute, Washington, DC 2011 http://pdf.wri.org/reefs_at_risk_revisited.pdf.

[3] Wilkinson C. Status of Coral Reefs of the World. Australian Institute of Marine Science, Townsville, Australia 2008 http://www.icriforum.org/sites/default/files/GCRMN_Status_Coral_Reefs_2008.pdf.

[4] Reaka-Kudla ML. The Global Biodiversity of Coral Reefs: A Comparison with Rain Forests In: Reaka-Kudla ML, Wilson DE, Wilson EO, editors. Biodiversity II: Understanding and protecting our biological resources. Washington DC: Joseph Henry Press; 1996 pp. 83–108.

[5] Mora C. Perpetual struggle for conservation in a crowded world and the needed paradigm shift for easing ultimate burdens In: Mora C, editor. Ecology of Fishes on Coral Reefs. Cambridge, United Kingdom: Cambridge University Press; 2015 pp. 289–296.

[6] Mora C, Aburto-Oropeza O, Bocos AA, Ayotte PM, Banks S, Bauman AG, et al Global human footprint on the linkage between diversity and ecosystem functioning in reef fishes. PLoS Biol. 2011; 9: e1000606 10.1371/journal.pbio.1000606 21483714 PMC3071368

[7] Pollock FJ, Lamb JB, Field SN, Heron SF, Schaffelke B, Shedrawi G, et al Sediment and turbidity associated with offshore dredging increase coral disease prevalence on nearby reefs. PLoS ONE. 2014; 9: e102498 10.1371/journal.pone.0102498 25029525 PMC4100925

[8] Hatcher BG, Johannes RE, Robertson AI. Review of research relevant to the conservation of shallow tropical marine ecosystems. Oceanogr Mar Biol Ann Rev. 1989; 27: 337–414.

[9] Erftemeijer PLA, Riegl B, Hoeksema BW, Todd PA. Environmental impacts of dredging and other sediment disturbances on corals: A review. Marine Poll Bull. 2012; 64: 1737–1765. 10.1016/j.marpolbul.2012.05.008 22682583

[10] Brock VE Van Heukelem W, Helfrich P. An Ecological Reconnaissance of Johnston Island and the Effects of Dredging. Hawaii Institute of Marine Biology Technical Reports No 11. 1966. http://hdl.handle.net/10125/15275.

[11] Islam A, Wang L, Smith C, Reddy S, Lewis A, Smith A. Evaluation of satellite remote sensing for operational monitoring of sediment plumes produced by dredging at Hay Point, Queensland, Australia. J Appl Remote Sens. 2007; 1: e011506 10.1117/1.2834768

[12] Roy KJ. Change in Bathymetric Configuration, Kaneohe Bay, Oahu, 1882–1969. Hawaii Institute of Geophysics Report. 1970; 70: 1–71. http://hdl.handle.net/10125/16312.

[13] Johannes RE. Pollution and degradation of coral reef communities In: Wood EJF, Johannes RE, editors. Tropical Marine Pollution. Amsterdam: Elsevier Scientific Publishing; 1975 pp. 13–50.

[14] Smith-Vaniz W, Collette BB, Luckhurst BE. Fishes of Bermuda. Lawrence, Kansas: Allen Press Incorporated; 1999.

[15] Dulvy NK, Sadovy Y, Reynolds JD. Extinction vulnerability in marine populations. Fish Fish. 2003; 4: 25–64.

[16] McManus JW, K. Shao T, Lin SY. Toward establishing a Spratly Islands international marine peace park: ecological importance and supportive collaborative activities with an emphasis on the role of Taiwan. Ocean Dev & Intl L. 2010; 41: 270–280.

[17] McManus JW, The Spratly Islands: A Marine Park? Ambio. 1994; 23: 181–186.

[18] Treml EA, Roberts J. Halpin PN, Possingham HP, Riginos C. The emergent geography of biophysical dispersal barriers across the Indo‐West Pacific. Divers Distrib. 2015; 21: 465–476.

[19] Mora C, Treml EA. Roberts J, Crosby K, Roy D, Tittensor DP. High connectivity among habitats precludes the relationship between dispersal and range size in tropical reef fishes. Ecography. 2011; 35: 89–96.

[20] Hughes TP, Huang H, Young MAL. The wicked problem of China's disappearing coral reefs. Conserv Biol. 2013; 27: 261–269. 10.1111/j.1523-1739.2012.01957.x 23140101

[21] Bruno JF, Selig ER. Regional decline of coral cover in the Indo-Pacific: timing, extent, and subregional comparisons. PLoS ONE. 2007; 2: e711 10.1371/journal.pone.0000711 17684557 PMC1933595

[22] Graham NAJ, Wilson SK, Jennings S, Polunin NVC, Bijoux JP, Robinson J. Dynamic fragility of oceanic coral reef ecosystems. Proc Natl Acad Sci USA. 2006; 103: 8425–8429. 16709673 10.1073/pnas.0600693103 PMC1482508

[23] Liu JY. Status of marine biodiversity of the China Seas. PLoS ONE. 2013; 8: e50719 10.1371/journal.pone.0050719 23320065 PMC3540058

[24] Huang D, Licuanan WY, Hoeksema BW, Chen CA, Ang PO, Huang H, et al Extraordinary diversity of reef corals in the South China Sea. Mar Biodiv. 2015; 45: 157–168.

[25] Wesseling I, Uychiaoco AJ, Aliño PM, Vermaat JE. Partial mortality in Porites corals: variation among Philippine reefs. Int Rev Hydrobiol. 2001; 86: 77–85.

[26] Wiens HJ. Atoll Environment and Ecology. New Haven: Yale University Press; 1962.

[27] Brown BE, Dunne RP. The environmental impact of coral mining on coral reefs in the Maldives. Environ Conserv. 1988; 15: 159–165.

[28] Ferrario F, Beck MW, Storlazzi CD, Micheli F, Shepard CC, Airoldi L. The effectiveness of coral reefs for coastal hazard risk reduction and adaptation. Nat Commun. 2014; 5: e3794 10.1038/ncomms4794 PMC4354160 24825660

[29] Kennedy EV, Perry CT, Halloran PR, Iglesias-Prieto R, Schonberg CHL, Wisshak M. Avoiding coral reef functional collapse requires local and global action. Curr Biol. 2013; 23: 912–918. 10.1016/j.cub.2013.04.020 23664976

[30] Zhao HT, Wu T. Some ideas about further development of the Xisha, Nansha and Zhongsha Islands. Trop Geog. 2008; 28: 369–375.

[31] White C, Costello C. Close the high seas to fishing? PLoS Biol. 2014; 12: e1001826 10.1371/journal.pbio.1001826 24667759 PMC3965379

