# Supplementary material for: Dredging in the Spratly Islands: Gaining Land but Losing Reefs
Source: PLoS Biol. 2016 Mar 31;14(3):e1002422. doi: 10.1371/journal.pbio.1002422 (PMC4816285; doi:10.1371/journal.pbio.1002422)
Supplement: S1 Data — (PDF) [file pbio.1002422.s001.pdf]

S1 Data: Methods used to quantify the area of reefs dredged and filled in the Spratly Islands using Landsat 8 imagery.

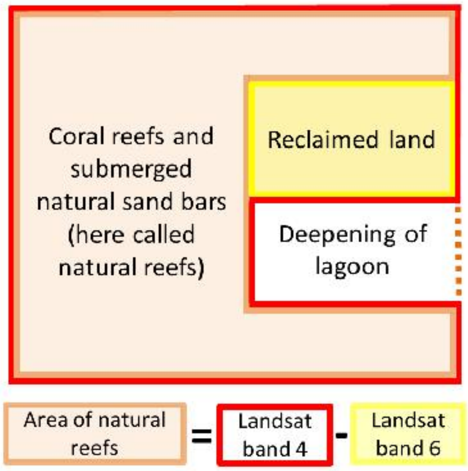

To quantify changes in reef and land areas for the seven atolls reclaimed by China, we extracted cloud-free Landsat 8 images (GeoTIFFs of bands 4 and band 6) from the United States Geological Survey website (http://landsat.usgs.gov/landsat8.php) for each 60 day period from February 2014 to May 2015. The dates and names of the images used are provided in S2 Data under the column headers “Date” and “Landsat Scene Identifier”, respectively. The sketch to the right and actual Landsat 8 images from Mischief Reef below (Credit: U.S. Geological Survey) illustrate how we calculated changes in total reef and land areas during the period of reclamation when images were available for all seven atolls.

Clarifications:

1. The area of coral reefs and submerged natural sand bars (hereafter natural reefs) and reclaimed lands is visible in the red optical band (i.e., Landsat 8 band 4; e.g. red arrows and polygons in images below and red-outline in the sketch above).
2. Only the area of reclaimed land is visible in the short wave infrared band (i.e., Landsat band 6; e.g. yellow arrows and polygons in the right-hand images below and yellow-outline in the sketch above). Neither coral reefs nor submerged natural sand bars are visible in band 6.
3. Atolls were largely submerged prior to the reclamation, with the exception of a few buildings standing upon pillars/columns over the reefs.

The area of natural reefs for each date was calculated by subtracting the size of the visible area in Landsat 8 band 6 (yellow outline in the sketch above) from the size of the visible area in Landsat 8 band 4 (red outline in the sketch above). The size of the area of reclaimed land is the area visible in Landsat 8 band 6 (yellow outline in the sketch above). Changes in these areas over the reclamation period are shown cumulatively for all atolls in Fig. 1C. Shapefiles of the polygons surrounding the visible areas in both Landsat 8 bands were created using ArcMap 10, and are available in S4 Data.

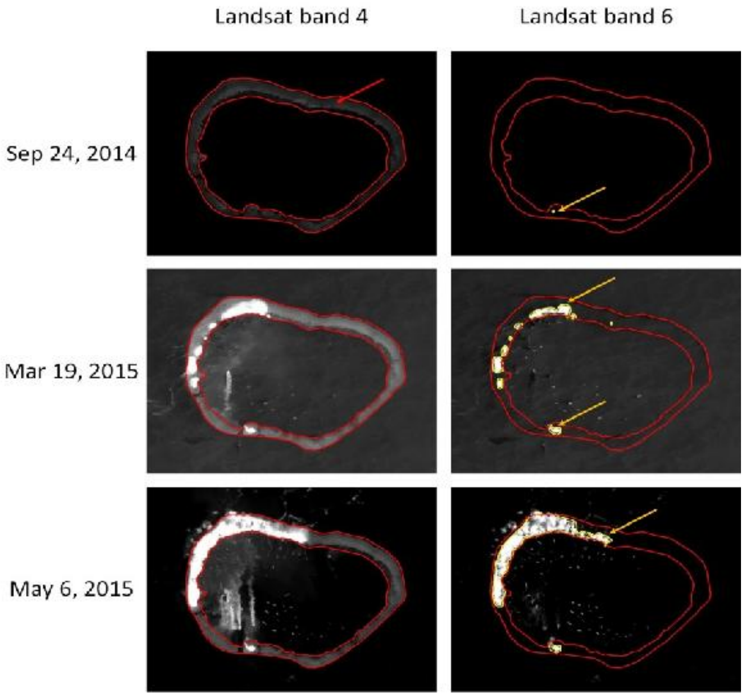

To calculate the cumulative changes in land and reef area, we first plotted the area of natural reefs and reclaimed land for each of the dates when cloud-free images were available for each atoll. We then used a cubic interpolation (using the “interp1” function of the R software package “pracma”) to connect the plotted points for each atoll. This was necessary before aggregation

because the dates available for each atoll differed. To calculate the aggregated areas of reclaimed land and natural reefs, we added the interpolated data for all of the atolls together for each date. Raw data indicating areas for individual atolls, interpolated data for each atoll, and aggregated data for all seven atoll are available in S2 Data.
